# Therapeutic uses of epicatechin in diabetes and cancer

**DOI:** 10.14202/vetworld.2017.869-872

**Published:** 2017-08-06

**Authors:** Layth Abdulmajeed Abdulkhaleq, Mohammed Abdulrazzaq Assi, Mohd Hezmee Mohd Noor, Rasedee Abdullah, Mohd Zamri Saad, Yun Hin Taufiq-Yap

**Affiliations:** 1 Department of Veterinary Laboratory Diagnostics, Faculty of Veterinary Medicine, Universiti Putra Malaysia, 43400 Serdang, Selangor, Malaysia; 2 Department of Pathology and Poultry Diseases, Faculty of Veterinary Medicine, Baghdad University, Baghdad, Iraq; 3 Department of Veterinary Preclinical Sciences, Faculty of Veterinary Medicine, Universiti Putra Malaysia, 43400 Serdang, Selangor, Malaysia; 4 Department of Community Health, College of Health and Medical Techniques, Al-Furat Al-Awsat Technical University, Iraq; 5 Department of Chemistry, Faculty of Science, Universiti Putra Malaysia, 43400 Serdang, Selangor, Malaysia

**Keywords:** angiogenesis, carcinoma, diabetes mellitus, epicatechin, oxidative stress

## Abstract

Epicatechin is a natural flavonoid found in green tea. It has been reported to possess an immense antioxidant effect which contributes to its therapeutic effect against a handful of ailments. In this review, we discuss its therapeutic role in the management of two of the most important human diseases; diabetes and cancer. The consumption of epicatechin has been shown to reduce blood glucose levels in diabetic patients, while is anticancer effect was attributed to its antioxidant properties, antiangiogenic and direct cytotoxicity to cancer cells. Although the exact mechanism of action of epicatechin is still being explored, there is no doubt that it is a promising candidate as an alternative. The significance of this review is to highlight the importance of the usage of natural products (in this case, epicatechin) as an alternative for the treatment of two potentially fatal diseases which is diabetes and cancer. The aim of this review is to educate the scientific community on the role of epicatechin in ameliorating the effects of diabetes and cancers on human while understanding the potential mechanisms of these aforementioned effects.

## Introduction

Epicatechin (flavon-3-ol monomer units) belongs to flavanol monomers which are subclass of flavonoids [1]. They are found in green tea [2], grape [3], and cocoa [4].

Rein *et al*. [5] have determined the antioxidant effect of different epicatechin concentrations in human plasma. The study showed that after 2 h of consumption, the serum levels of epicatechin increased to 12-fold while the capacity of its mean plasma antioxidant increased considerable to 40% higher than the original untreated human [5]. This flavanol had improved functions of human endothelial cells [6].

Another published work suggested that the human subjects who consumed food containing <50 mg epicatechin exhibited a great elevation in epicatechin plasma concentrations as well as increased endothelium-dependent flow-mediated dilation [7]. The elevations in serum epicatechin concentrations led to increased flow-mediated dilation [8]. The increased of epicatechin concentrations extracted from many foods was responsible for the elevated endothelium-derived vasodilatation [9]. On the other hand, it must be mentioned that it is undefined how epicatechin may interact with physiological and biochemical systems in human body to produce these findings.

The aggregations of platelet which can be induced as a result to their increased activity cause various coronary artery diseases [10]. A lot of researchers have demonstrated that the epicatechin rich green tea can exhibit *in vivo* platelet antiaggregation effect as determined in murine and human [11].

The *in vitro* exposure of cells by cacao (rich in epicatechin) led to significant inhibition of epinephrine-induced platelet induction [12]. The consumption of cacao (containing 200 mg of epicatechin) has inhibited the platelet induction in human body [13].

Another study validated these results as the collagen-activated platelet aggregation were inhibited significantly in platelet of rat exposed to *Bulnesia sarmientoi* aqueous extract (which is rich in epicatechin), in that way it decreases the possibility of developing cardiovascular disease [14].

### Epicatechin with Insulin Sensitivity

The resistance generated against insulin receptors reduces insulin uptake which leads to accumulations of glucose in the blood circulation is known as type II diabetes [15]. The cardiovascular diseases development by insulin sensitivity (as an independent risk factor) is still under debate [16]. A study by Cremonini *et al*. [17] has revealed the effects of epicatechin on insulin sensitivity.

A study by Josic *et al*. [18], has revealed the effects of epicatechin on insulin sensitivity, stated that the consumption epicatechin rich green tea could be reduced glucose and oral testing insulin values, in addition to reduced glucose and fasting insulin concentrations [18]. Moreover, epicatechin displayed greater quantitative insulin sensitivity and a lower homeostasis model assessment of insulin resistance and had no effect on fasting plasma glucose. Epicatechin did not change blood pressure [19]. Blood pressure showed that epicatechin consumption decreased the systolic blood pressure. Epicatechin showed no significant effects on the diastolic blood pressure values [20].

In another study conducted by Cremonini *et al*. [17], suggested that epicatechin has been found to elevate insulin sensitivity as well as to lower insulin resistance [17]. It is well-know that the increase in uptake of nitric oxide normally occurred in endothelial cells and this lead to decrease in blood pressure [17]. Therefore, it can be assumed that the insulin sensitivity following epicatechin consumption is attributed to decrease in the systolic blood pressure even though the nitric oxide release was not quantified [21].

Nevertheless, epicatechin consumption has attributed toward various cellular mechanisms including adjusted insulin sensitivity [22]. Epicatechin present in green tea and chocolate displayed decrease in both insulin resistance and blood pressure. Through reducing both insulin resistance and blood pressure, consumption of epicatechin–containing food can help prevent the onset of type II diabetes and many cardiovascular diseases [11,23].

### Epicatechin with Oxidative Stress

Inflammation and oxidative stress represent common responses which may contribute in the development of tumor via stimulating defected cells to go through promotion and progression of tumors, initiating direct damage to genomic nucleic acids, initiating abnormal cell growth and modifying intracellular signaling [24]. The immune cells are supported by some pathways of protecting against oxidative stress products as well as defending from free radicals released such as reactive oxygen species [25].

Reactive oxygen species and other free radical species are being found to be a main cause for the inception of oxidative stress, and in this manner leads to cellular damage [26], which may subsequently cause carcinogenesis [27].

Efficacy of reactive oxygen species and related radicals (e.g. peroxynitrite) had been demonstrated due to their potency to stimulate oxidative stress which can be induced by inflammation [28]. A study conducted by Xiong [28] has revealed that the epicatechin and related isomers have restrained oxidative responses including peroxynitrite. The epicatechin tetramer has exhibited greater chemopreventive effect against oxidative damage than seen with epicatechin alone [29].

### Epicatechin with Angiogenesis and Cancer

The development of new blood capillaries which formed from neighboring vessels is a process known as angiogenesis [30]. Since the new capillaries are developed with tumor growth with the purpose of supplying nutrients and excreting cellular wastes, angiogenesis is always associated with cancer formation [31]. The discontinuing capacity of new capillaries to form, the potency of cancer cells to grow and develop would also be suppressed [32,33].

The effects of epicatechin on the rapid increase in numbers of human dermal microvascular endothelial cells after angiogenic induction have been investigated [34]. It has been confirmed that epicatechin suppressed proliferation of induced human dermal microvascular endothelial cells [34]. Moreover, the epicatechin and related compounds have been found to modulate the signaling enzymes activity during angiogenic signaling, controlling its releasing and up-regulating its receptors [34].

Studies conducted by some researchers indicate that epicatechin may have a great influence on factors related to cancer metastasis [35]. Other researchers noted that the dose of epicatechin used in the *in vitro* work most likely goes above the concentration observed *in vivo* [35]. Their results on angiogenesis regulation encouraged deeper evaluations involving many genes related to cellular signaling.

### Epicatechin Inhibition of Human Cancer Cell Growth

The effects of green tea and epicatechin rich extracts on the growth of many cancer cell lines were studied [36]. Findings demonstrated that the green tea and epicatechin rich extracts inhibited proliferation of cancer cells in a concentration-dependent manner. Epicatechin extracted from green tea with a concentration of 100 µM revealed the slightest inhibition against cervical cancer Hela cells and gastric adenocarcinoma MKN-45 cells [37].

However, extractions from green tea containing 140 mg/g of epicatechin and related compounds demonstrated a 20% growth inhibition of human bladder cancer TCCSUP cell line at 20 µg/mL [38]. Necrotic cell death was also detected from the epicatechin rich extracts. The key enzyme activities that include H_2_O_2_-mediated oxidative stress can be increased to aid in destruction of rapid spread of cancer cells [38].

These finding demonstrated that epicatechin rich extracts in high doses have *in vitro* antiproliferative potency. Many related flavanols displayed prevent human cancer cells proliferation [38].

Epicatechin is a flavonoid found in dark chocolate harvested from the cacao tree. Epicatechin has been demonstrated in animals and humans to increase the production of new mitochondria in heart and muscle (termed “mitochondrial biogenesis”) while concurrently stimulating the regeneration of muscle tissue. Some researchers show for the first time that a single oral daily dose of (-)-epicatechin (2 or 10 mg/kg) partially or fully prevented most of the effects induced by L-nitroarginine methyl ester (L-NAME) nitroarginine, is a nitro derivative of the amino acid arginine. It is an inhibitor of nitric oxide synthase and hence a vasoconstrictor. As such, it finds widespread use as a biochemical tool in the study of nitric oxide and its biological effects such as (a) increases in the left ventricular hypertrophy, (b) proteinuria, (c) renal histological lesions, (d) increased plasma malondialdehyde concentrations and urinary iso-PGF2a excretion, (e) increased endothelium-dependent contraction and cyclooxygenase-2 overexpression, (f) increased vascular production of O^−^_2_ and nicotinamide adenine dinucleotide phosphate (NADPH) oxidase activity, and (g) increased vascular inflammatory status. In most cases, these effects were dose-dependent. However, it did not inhibit the development of hypertension and had only minor effects on the impaired endothelium- and nitric oxide (NO)-dependent relaxation. Interestingly, changes in several end-points were not dependent on the presence of (-)-epicatechin in blood, since they were obtained 48 h after the deprivation of the flavanol, indicating that it alters the course of the disease via permanent structural changes and/or alteration of gene expression. (-)-Epicatechin, at concentrations. 30 mM, exhibits vasodilator effects *in vitro*, which are partially endothelium- and NO-dependent [39].

(-)-Epicatechin activates endothelial NOS (eNOS), also known as nitric oxide synthase 3 (NOS3) or constitutive NOS, is an enzyme that in humans are encoded by the NOS3 gene located in the 7q35-7q36 region of chromosome 7. Therefore, a functional eNOS is essential for a healthy cardiovascular system in human coronary artery endothelial cells by (i) Ser-633 and Ser-1170 phosphorylation and Thr-495 dephosphorylation, and (ii) via Ca_2_/calmodulin-dependent kinase II pathways, leading to increase NO production [40]. Moreover, (-)-epicatechin and its two *in situ* O-methylated metabolites elevate NO in endothelial cells via inhibition of NADPH oxidase [41].

(-)-epicatechin also increased Akt and eNOS phosphorylation and prevented the L-NAME-induced increase in systemic (plasma malondialdehyde and urinary 8-iso-PGF2a) and vascular (dihydroethidium staining, NADPH oxidase activity and p22phox up-regulation) oxidative stress, proinflammatory status (intercellular adhesion molecule-1, interleukin-1b, and tumor necrosis factor-alpha up-regulation), and extracellular signal-regulated kinase 1/2 phosphorylation [42]. (-)-Epicatechin *in vitro* induced activation of eNOS through two mechanisms: (i) eNOS phosphorylation by the participation of the phosphatidylinositol 3-kinase pathway and (ii) activation of the Ca_2_/calmodulin-dependent kinase II pathway [40].

## Conclusion

Epicatechin is one of the many abundant flavonoids in nature. This review has highlighted its beneficial role in the reduction of blood glucose levels in diabetic patients. Its anticancer effect has also been discussed to entail its antioxidant, antiangiogenic, and antiproliferative effects against cancer cells. The high safety margin of epicatechin contributed to its therapeutic successes in the management of diabetes and cancer. However, its exact mechanism of action in these two conditions is still being explored.

## Authors’ Contributions

All authors mentioned have made substantial contribution to this manuscript and approved the list of authors in this review. All authors critically revised and contributed to the intellectual content, and also editing of the paper. They all approved the final draft for submission and the list of authors.
